# Rituximab Biosimilar BCD020 Shows Superior Efficacy above Conventional Non-Biologics Treatment in Pediatric Lupus Nephritis: The Data of Retrospective Cohort Study

**DOI:** 10.3390/biomedicines11051503

**Published:** 2023-05-22

**Authors:** Mikhail Kostik, Elvira Kalashnikova, Raupov Rinat, Eugenia Isupova, Ekaterina Gaidar, Anton A. Soloviev, Vera Masalova, Ludmila Snegireva, Tatyana Kornishina, Natalia Abramova, Evgeny Suspitsin, Lubov Sorokina, Maria Kaneva, Margarita F. Dubko, Natalia Lubimova, Ekaterina Kuchuinskaya, Olga Kalashnikova, Vyacheslav Chasnyk

**Affiliations:** 1 Hospital Pediatry, Saint-Petersburg State Pediatric Medical University, 194100 Saint Petersburg, Russiarinatraup94@gmail.com (R.R.); solant8@gmail.com (A.A.S.); evgeny.suspitsin@gmail.com (E.S.); chasnyk@gmail.com (V.C.); 2 Almazov National Medical Research Centre, 197341 Saint Petersburg, Russiakuchinskaya.link@gmail.com (E.K.); 3 N.N. Petrov National Research Center of Oncology, 197758 Saint Petersburg, Russia

**Keywords:** systemic lupus erythematosus, B-cells, rituximab, children, lupus nephritis

## Abstract

Background: Pediatric lupus nephritis (LN) is one of the most serious manifestations of systemic lupus erythematosus (SLE) in children, determining the outcomes of the disease. There are no standardized treatment protocols for pediatric LN, and the role of biologics has not yet been conclusively defined. Objectives: analyze the safety and efficacy of rituximab biosimilar BCD020 in pediatric patients with lupus nephritis. Methods: in a retrospective cohort study, the data from the case histories of 25 patients with LN (10 boys and 15 girls) with an onset age of 13 (9–16) years, who failed conventional non-biologic treatment or developed corticosteroid dependence/toxicity, were included. The diagnosis was made using Systemic Lupus International Collaborating Clinics (SLICC) classification criteria. Rituximab biosimilar BCD020 was prescribed in a dosage of 375 mg/m^2^ every week (2–4 infusions) with repeated courses every 6–12 months (2–4 infusions) according to disease activity, B-cell depletion, and IgG levels. The dynamics of clinical and laboratory data, the activity of the disease by SLEDAI, and corticosteroid doses were assessed at the onset and during the rituximab trial. Results: The main patient’s characteristics were: Pre-rituximab non-biologic conventional treatment included: cyclophosphamide 15 (60%), MMF 8 (32%), azathioprine 3 (12%), hydroxychloroquine 12 (48%), and pulse therapy of methylprednisolone followed by oral methylprednisolone 25 (100%). The time before rituximab was 7.0 (3.0–24.0) months, and the whole observation period was 7.0 (0; 24) months. The initial pre-rituximab treatment slightly reduced SLEDAI levels and the proportion of patients with LN. A significant reduction of SLEDAI, the anti-dsDNA level, proteinuria, hematuria, C4 complement, ESR, and the median corticosteroid dose by 80% from the initial value, as well as the proportion of patients without corticosteroids, was observed after rituximab administration. Two deaths were observed due to catastrophic SLE with macrophage activation syndrome, accompanied by a severe infection (invasive aspergillosis, *n* = 2). Three patients developed serious adverse events: pneumonia (*n* = 2), transient agranulocytosis (*n* = 1) after the third rituximab infusion, and meningitis, caused by Listeria monocytosis, after the first rituximab infusion. Eight patients received antibacterial treatment for different respiratory infections without hospital admissions. Conclusions: Rituximab biosimilar BCD020 showed effectiveness in LN, whereas previous non-biologic treatment was insufficiently effective. Randomized controlled trials are required to evaluate the efficacy and safety of rituximab and evaluate the benefits when compared with conventional SLE treatment.

## 1. Introduction

Juvenile systemic lupus erythematosus (SLE) is the most common connective tissue disease in children under 18 years of age, characterized by multi-organ involvement and a serious prognosis [1]. SLE in adolescents and young adults is characterized by a more active course with more severe damage to various organs and systems compared to adult patients [1,2]. The severity of SLE in young patients is related to a higher frequency of kidney and central nervous system (CNS) involvement as well as hematological manifestations [3].

Corticosteroids remain the basis of therapy for SLE and lupus nephritis (LN) [2]. The toxicity of corticosteroids and their long-term use requires steroid-sparing therapy administration [4,5,6]. Currently, a consensus has been reached on the treatment of lupus nephritis in children, along with recommendations for reducing corticosteroid therapy [7]. Despite EULAR recommendations on minimizing corticosteroid therapy, standardized recommendations on the rate of reduction and withdrawal of corticosteroids, except for lupus nephritis, have not been developed, especially in pediatrics [8,9]. Standard-of-care treatment (SOCT) with cytostatics (e.g., azathioprine, mycophenolate mofetil, cyclophosphamide) is accompanied by various adverse events on hematopoiesis, liver and kidney toxicity, increased risk of infertility, and a delayed risk of malignant neoplasms in adults compared with a healthy population [3,8].

The use of biologic therapy has significantly modified the course and outcomes of many rheumatic diseases, such as rheumatoid arthritis, ankylosing spondylitis, psoriatic arthritis, juvenile idiopathic arthritis, ANCA-associated vasculitis, and aortoarteritis [10]. For the treatment of SLE, two biological drugs are currently used: rituximab and belimumab. Belimumab is approved in adults and children over 5 years of age and can be used in patients with a moderately severe disease [11,12]. There are no data about the efficacy of belimumab in severe, life-threatening, highly active SLE. Belimumab rather acts as a steroid-sparing agent in cases involving a corticosteroid-dependent course of SLE or the maintenance of a previously achieved remission, especially in light of the strategy of “corticosteroid-free” therapy of SLE [3,6].

Rituximab is used to treat severe, life-threatening variants of SLE, but it has not had official approval for SLE in adults and children. Rituximab is a chimeric mouse/human IgG1-k monoclonal antibody with an affinity to the surface CD-20 antigen of B-lymphocytes, causing the depletion of B-cells and preventing the further formation of plasma cells, the production of auto-antibodies, and intercellular cooperation with B-lymphocytes [1,13]. Rituximab is considered an option in cases of high-activity SLE with the involvement of kidneys, the central nervous system, and hematological manifestations in case of failure of the standard-of-care treatment [3,6,14]. However, the position of rituximab as a first-line therapy in combination with corticosteroids and non-biological disease-modifying anti-rheumatic drugs (DMARD) remains open and requires more evidence of rituximab efficacy and safety [5,6]. Despite the known clinical efficacy of the drug, several retrospective comparative studies have not shown the advantages of rituximab compared to traditional non-biologic DMARD, and no prospective placebo-controlled studies have been conducted in comparison with SOCT in children with LN [15,16,17]. The present study aimed to evaluate the safety and efficacy of rituximab biosimilar BCD020 in pediatric patients with LN when compared to the previous conventional non-biologic treatment.

## 2. Materials and Methods

### 2.1. Study Design

In this retrospective cohort study, information on 25 patients with LN (10 boys and 15 girls) who failed conventional non-biologic treatment or developed corticosteroid dependence/toxicity from two centers were included. Diagnosis of SLE was made using Systemic Lupus International Collaborating Clinics (SLICC) classification criteria [18]. Lupus nephritis was diagnosed according to the criteria of the International Society of Nephrology/Renal Pathology Society [19]. The observation period was 2014–2021 years.

Indications for rituximab were: (i) a high active disease course resistant to non-biologic conventional treatment; and (ii) the presence of corticosteroid toxicity or impossibility to reduce corticosteroids to 10 mg/day or 0.2 mg/kg/day. Rituximab biosimilar BCD020 was prescribed in a dosage of 375 mg/m^2^ every week (2–4 infusions) with repeated courses every 6–12 months (2–4 infusions) according to the disease activity, B-cell depletion, and IgG levels.

### 2.2. Assessment

In each patient, we evaluated the dynamics of all available parameters of SLE and LN, treatment before and during rituximab treatment in three-time points: (i) disease onset; (ii) before rituximab treatment; and (iii) last available visit in the clinic closer to 18 years:

- demography: gender, onset age, family history positive for SLE, the type of LN;
- disease activity: the levels of antinuclear antibodies, antibodies to double-stained DNA, C3, C4, hemoglobin, platelets, complete blood count, ESR, CRP, urea, creatinine, serum protein and albumin, proteinuria, presence of leucocyturia and hematuria, SLEDAI [18] and LN activity stage, B-cell level, and IgG;
- concomitant treatment: the presence of corticosteroids and median dose;
- hydroxychloroquine;
- non-biologic DMARD;
- adverse events.

### 2.3. Outcomes

- the number of patients with inactive LN;
- the dynamics of proteinuria, hematuria;
- the dynamics of SLE activity: SLEDAI, anti-dsDNA level, C4 complement, ESR;
- the dynamics of oral corticosteroid dose;
- adverse events.

### 2.4. Statistics

The statistical analysis was performed with the software STATISTICA, version 10.0 (StatSoft Inc., Tulsa, OK, USA). All continuous variables were checked by the Kolmogorov–Smirnov test: no normal distribution was identified. Continuous variables are presented as median and interquartile ranges (IQRs). Categorical variables are presented as proportions. Missing data were not imputed or included in the analysis. Pearson’s χ^2^ test or Fisher’s exact test in the expected frequencies <5 was used to compare the categorical variables. A comparison of two dependent quantitative variables was carried out using Wilcoxon’s matched paired test and the Mac-Nemar test was applied for dependent categorical variables. A *p*-value of less than 0.05 was considered statistically significant.

### 2.5. Pre-Rituximab Patients’ Characteristics

The main patient’s characteristics were: pre-rituximab non-biologic conventional treatment included cyclophosphamide 15 (60%), mofetil mycophenolate (MMF) 8 (32%), azathioprine 3 (12%), hydroxychloroquine 12 (48%), and pulse therapy of methylprednisolone followed by oral methylprednisolone 25 (100%). Kidney biopsy was performed in 12 (48%) patients with LN, and the majority of patients had a III/IV class of LN. Two patients had low and early-developed ESRD and received hemodialysis. All patients received initial non-biologic standard-of-care treatment. Patients’ initial characteristics are presented in Table 1.

### 2.6. Pre-Rituximab Treatment Efficacy

The time before rituximab was 7.0 (3.0; 24.0) months. Initial pre-rituximab treatment slightly reduced the SLEDAI levels (−22%) and the proportion of patients with LN (−40%). The median daily dose of corticosteroids was reduced by 20%. The anti-dsDNA level decreased by 76% but did not reach the level of significance.

## 3. Results

### 3.1. The Rituximab Treatment

The duration of the rituximab part of the study ranged from 6 months to 3 years, and the median duration was 8 months (2; 32). We observed a prominent reduction of SLEDAI (−60%), the anti-dsDNA level (−16%), proteinuria, hematuria, C4 complement, ESR, and the median corticosteroid dose by 60%, as well as the proportion of patients without corticosteroids (−36%) following rituximab administration. Renal function was improved, the GFR increased, and hemodialysis was interrupted in patients who previously required it. The data are shown in Table 2.

### 3.2. Safety of Rituximab Treatment and Infection Prophylaxis

There were no severe infusion reactions that required the discontinuation of the infusion. Two deaths were observed due to catastrophic SLE with MAS, accompanied by a severe infection (invasive aspergillosis, *n* = 2). Three patients had SAE: pneumonia (*n* = 2), transient agranulocytosis (*n* = 1) after the third rituximab infusion, and meningitis, caused by Listeria monocytogenis, after the first rituximab infusion. This patient received two additional courses of rituximab eight months after the meningitis recovery without any following adverse events. Eight patients received antibacterial treatment for different respiratory infections without hospital admissions. The majority of infectious events were observed in the first year of the rituximab treatment. The majority of patients received cotrimoxazole prophylaxis. Five patients, who developed SAE, received IVIG (including patients with macrophage activation syndrome). Six patients with hypogammaglobulinemia (IgG < 4.5 g/L) received replacement IVIG treatment for infection prophylaxis.

## 4. Discussion

Our study demonstrated the efficacy and safety of rituximab in patients with LN who initially did not respond to SOCT or who had signs of corticosteroid toxicity. Similar to our study, the main indication for rituximab according to the literature was a failure of corticosteroid and cytostatic therapy in severe cases of SLE [6,14,16,17]. Despite many years of experience in the treatment of SLE, rituximab has not yet received official approval in either adults or pediatric practice due to the lack of randomized clinical trials confirming the efficacy of rituximab in SLE [14]. Numerous cohort studies, case series, and clinical cases have shown the effectiveness of rituximab in patients with SLE with varying degrees of activity, including with catastrophic courses, which, together with expert opinion, allowed rituximab to be introduced into the algorithms of SLE therapy for both children and adults [6,7,10].

Previous studies have demonstrated a positive effect of rituximab in SLE in both adults and children, including in the treatment of LN in adults and children, with an efficacy rate of up to 100% [1,4,5,17,20,21,22,23]. The remission of LN was achieved in an additional 28% of our cohort, and improvement in LN was observed in all patients. No significant flares were observed during the observation period, similar to other studies [4,5]. The most important outcomes of rituximab were the rapid and highly effective control of lupus nephritis and a significant reduction in the dose of corticosteroids without disease progression [1,4,5,21,22,23]. The superiority of rituximab over SOCT in pediatric LN patients was shown in one study [5].

In addition to increasing the proportion of patients with inactive LN, improving renal function, and reducing proteinuria and hematuria, there was a decrease in SLE activity markers, such as SELENA-SLEDAI and the levels of antibodies to double-stained DNA, similar to the results of other pediatric studies [4,21,22,23].

A hematological improvement, normalization of the complement level, and decrease in the levels of antibodies to double-stained DNA were observed during rituximab treatment in several previous studies, similar to our results [21,22,23]. A significant reduction in the dose of corticosteroids during the rituximab trial, as a result of a decrease in the activity of the disease, was demonstrated both in our study and in previously published studies [1,4,5,21,22,23]. The most frequent adverse events of rituximab were deaths, infusion reactions with fever, malaise, and headache [1,4,21,22,23]. The rate of severe adverse events ranged from zero [4] to nearly half the patients [1].

Some authors reported a similar rate of adverse events in non-rituximab patients [21], or less than in LN patients, treated with cyclophosphamide [5]. Cytopenia was noted among non-infectious complications [1,21]. Among infectious complications, there are serious cases, such as septicemia, endocarditis caused by Staphylococcus aureus, and milder infections, e.g., herpes labialis, herpes zoster, candidiasis, and chickenpox [1,4,5,21,22,23]. There are studies in which infectious complications in some patients receiving rituximab therapy were not noted, despite hypogammaglobulinemia [1,4,21].

Polymorphism of clinical manifestations of SLE is one of the factors that complicate the standardization of patients and therapy, which significantly complicates the conducting of randomized clinical trials [3,16,17].

### Study Limitations

The limitations of our study are related to its retrospective nature, missing data, the lack of a unified treatment protocol, and different LN classes of patients. Often, the choice of drug, changes in the treatment, indications for rituximab, and time of initiation are based on the personal opinion/experience of the attending physician, which could affect the results of the study. The assessment of the real rate of SAE is also difficult to calculate due to the small sample size and use of rituximab in specific pediatric populations with severe catastrophic courses of SLE.

## 5. Conclusions

The implementation of rituximab biosimilar BCD020 therapy allowed for a more effective treatment of SLE patients and the minimization of the side effects of standard therapy. It is necessary to evaluate the effectiveness of rituximab not only as a rescue treatment in cases of severe courses of SLE and the failure of standard therapy, but also as a remission induction tool in forms of SLE that are not only severe but also moderate, for which it is necessary to conduct prospective placebo-controlled trials and compare the effect of rituximab therapy with SOCT, while including a long follow-up observation period.

## Figures and Tables

**Table 1:** Characteristics of patients at the time of the disease’s onset.

Clinical Features	*n* (%)	Laboratory Features	*n* (%)
Onset age, years, Me (min–max)	13.0 (9.0; 16.0)	Antinuclear antibodies positivity, *n* (%)	24 (96)
Sex, girls, *n* (%)	15 (60)	Anti-dsDNA, U/mL, Me (25%; 75%)	150 (48; 237)
Fever, *n*, (%)	18 (72)	Anti-dsDNA antibodies, *n* (%)	20 (80)
Skin involvement, *n*, (%)	23 (92)	Erythrocyte sedimentation rate, mm/h, Me (25%; 75%)	21 (9; 32)
Oral mucosa involvement, *n*, (%)	7 (28)	C-reactive protein, mg/L, Me (25%; 75%)	1.7 (0.8; 6.7)
Alopecia, *n*, (%)	2 (8)	Antiphospholipid antibodies, *n*, (%)	8 (32)
Arthritis, *n*, (%)	15 (60)	Coomb’s positivity, *n*, (%)	12 (48)
Central nervous system involvement, *n*, (%)	13 (52)	Complement, C3 (n.v. 0.9–1.8), g/L, Me (25%; 75%)	0.64 (0.44; 1.23)
Pleuritis, *n*, (%)	10 (40)	Complement, C4 (n.v. 0.1–0.4), g/L, Me (25%; 75%)	0.11 (0.06; 0.38)
Pericarditis, *n*, (%)	8 (32)	Hemoglobine, g/L, Me (25%; 75%)	98 (91; 121)
Ascitis, *n*, (%)	10 (40)	Anemia, *n* (%)	16 (64)
Myocarditis, *n*, (%)	4 (16)	Platelets ×10^9^/L, Me (25%; 75%)	269 (181; 320)
Lung involvement, *n* (%)	5 (20)	Thrombocytopenia, *n*, (%)	8 (32)
Hepatomegaly, *n*, (%)	10 (40)	White blood cells ×10^9^/L, Me (25%; 75%)	6.7 (4.5; 9.0)
Splenomegaly, *n*, (%)	3 (12)	Leucopenia, *n*, (%)	17 (68)
Lymphadenopathy, *n*, (%)	4 (16)	Proteinuria, *n*, (%)	25 (100)
Gastrointestinal involvement, *n*, (%)	4 (16)	Proteinuria, g/L	2.4 (1.3; 8.7)
Sicca syndrome, *n*, (%)	2 (8)	Hematuria, cells	15 (0; 42)
Skin vasculitis, *n*, (%)	8 (32)	Hematuria, *n*, (%)	16 (64)
Palmar erythema, *n*, (%)	4 (16)	Urea, mmol/L, Me (25%; 75%)	9.1 (4.2; 11.2)
Raynaud’s syndrome, *n*, (%)	2 (8)	Creatinine, mkmol/L, Me (25%; 75%)	68 (52; 142)
Thrombosis, *n*, (%)	4 (16)	Low glomerular filtration rate, *n*, (%)	3 (12)
Macrophage activation syndrome, *n*, (%)	6 (24)	End-stage renal disease, *n* (%)	2 (8)
SLEDAI onset, Me (25%; 75%)	23 (16; 26)	Kidney biopsy, *n*, (%)	12 (48)
SLEDAI onset, grade, *n* (%)		Lupus nephritis, class, *n* (%)	
–5 points	0 (0)	II *n*, (%)	1 (8)
6–10 points	2 (8)	III *n*, (%)	3 (25)
11–19 points	9 (36)	IV *n*, (%)	7 (59)
>20 points	14 (56)	V *n*, (%)	1 (8)
**Treatment**			
Intravenous corticosteroids, *n* (%)	20 (80)	DMARDS, *n* (%)	
Oral corticosteroids, *n* (%)	24 (96)	Cyclophosphamide	15 (60)
Oral corticosteroids, mg/kg	1.0 (0.7; 1.0)	Mofetyl mycophenolate	8 (32)
Hydroxichloquine, *n* (%)	12 (48)	Azathioprine	3 (12)

Abbreviations: anti-dsDNA—antibodies to double-stained DNA; DMARDs—disease-modifying anti-rheumatic drugs; Me—median; n.v.—normal value.

**Table 2:** The dynamics of SLE and LN features before and under rituximab treatment.

Parameter	SLE Onset	RTX (Baseline)	p_1_	LV	p_2_	p_3_
SLEDAI, Me (IQR)	23 (16.0; 26)	18.0 (8,0; 26,0)	0.003	4 (2.0; 8.0)	0.0015	0.000001
ANA, titer, Me (IQR)	5120 (1280; 10240)	1280 (240; 3200)	0.345	640 (160; 1280)	0.515	0.0014
Anti-dsDNA, U/mL (n.v. < 25), Me (IQR)	150 (48; 237)	36 (7.7; 148)	0.263	12 (0; 65)	0.05	0.001
Proteinuria, g/L, Me (IQR)	1.0 (0.5; 3,5)	0.6 (0.3; 3.7)	1.0	0.17 (0; 0.9)	0.05	0.0008
Hematuria, #cells, Me (IQR)	15 (0; 42)	15 (5; 50)	0.170	0 (0; 2,5)	0.088	0.003
Complement, C4, g/L, Me (IQR)	0.11 (0.06; 0.4)	0.13 (0.06; 0.24)	0.715	0.16 (0.1; 0.2)	0.042	0.0066
ESR, mm/h, Me (IQR)	21 (9; 32)	17 (9; 26)	0.610	12 (3; 21)	0.023	0.014
Patients with active LN *n*, (%)	25 (100)	15 (60.0)	0.001	8 (32.0)	0.048	0.001
Patients on GCS therapy *n*, (%)	25 (100)	25 (100)	1.0	16 (64)	0.005	0.004
Glucocorticosteroids, mg/kg, Me (IQR)	1.0 (0.7; 1.0)	0.8 (0.23; 1.0)	0.148	0.2 (0.1; 0.9)	0.001	0.0002

Abbreviations: ANA—antinuclear antibody, ESR—erythrocyte sedimentation rate, GCS—glucocorticosteroids, LN—lupus nephritis, LV—last visit, RTX—rituximab, SLE—systemic lupus erythematosus. p_1_ comparison between onset and RTX baseline, p_2_—comparison between RTX baseline and LV, p_3_—comparison throughout the study.

## Data Availability

The datasets generated during the current study are available from the corresponding author upon reasonable request.
